# New Hopes for Drugs against COVID-19 Come from the Sea

**DOI:** 10.3390/md19020104

**Published:** 2021-02-11

**Authors:** Orazio Taglialatela-Scafati

**Affiliations:** Department of Pharmacy, School of Medicine and Surgery, University of Naples Federico II, Via D. Montesano 49, I-80131 Naples, Italy; scatagli@unina.it; Tel.: +39-081678509

The latest chapter of the historic battle of humans against pathogenic microbes is the severe acute respiratory syndrome (SARS)-like coronavirus-2 (SARS-CoV-2), responsible for COVID-19, a respiratory disease declared a global pandemic by the WHO on March 11, 2020. Less than one year later, at the beginning of February 2021, nearly 104 million cases and 2.3 million deaths have been reported worldwide [1], but the effects on the global economy (and on our psychological wellbeing) will probably last for years.

The progressive approval and utilization of effective vaccines, including two unprecedented mRNA-based ones, is very good news that was largely unexpected only a few months ago, and that could allow us to watch with a cautious optimism to the months to come. However, the full control of COVID-19 cannot rely only on vaccines; it will also require efficacious therapeutics, which could possibly be used to face future related infections more efficiently. Although several molecules are currently undergoing clinical trials, the single FDA-approved drug is remdesivir [2], alone or in combination with baricitinib, indicated to treat severe COVID-19 cases in adults and children (≥12 years old).

Repurposing existing drugs has been a very popular strategy among scientists searching for possible COVID-19 treatments, especially those trying to block the recognition between the SARS-CoV-2 spike glycoprotein and the cellular angiotensin-converting enzyme 2 (ACE2). Additionally, many anti-inflammatory and anticancer drugs have been repurposed to inhibit the massive inflammatory response (the so-called *cytokine storm*) resulting from the SARS-CoV-2 infection.

In the frame of this global endeavor, the contribution of researchers working on marine natural compounds has been very significant. Kim et al. have reported [3] that lambda-carrageenan (λ-CGN), sulfated galactose-based polysaccharides purified from marine red algae, efficiently inhibited SARS-CoV-2 with a submicromolar activity, reducing the expression of viral proteins in cell lysates and suppressing progeny virus production in culture supernatants. Most likely, these polymeric compounds act by targeting viral attachments to cell surface receptors, thus preventing virus entry.

*Marine Drugs* has published six articles on marine molecules with potential against coronavirus, including three review articles [4,5,6] and three research papers [7,8,9]. In two of these [7,8], the authors suggest that another marine natural polymer, the inorganic polyP, abundantly present in marine bacteria, is worthy of further investigation for its activity in strengthening the mucin barrier and inhibiting viral attachment to the cells.

Another very promising result has been reported in a preprint paper by Gerwick, O’Donoghue and Payne [10]. They have identified the marine cyanobacterial depsipeptide gallinamide A/symplostatin 4 (**1**, Figure 1) and some synthetic analogues as potent (in the picomolar range) and selective inhibitors of cathepsin L. This lysosomal cysteine protease is utilized by coronaviruses to release RNA material inside the cell and, consequently, its blockade results in a marked inhibition of SARS-CoV-2 infection in vitro.

However, probably the most exciting discovery was published at the end of January 2021 in *Science* [11]. White, Rosales et al. have reported that, in studies in human cells, plitidepsin (dehydrodidemnin B, **2**), a depsipeptide originally isolated from the tunicate *Aplidium albicans* and marketed with the name Aplidin^®^ by Pharmamar, largely outperformed remdesivir against SARS-CoV-2. Plitidepsin target is the human protein eEF1A, whose expression is related to cancer insurgence, but which is also involved in the interaction with the coronavirus nucleocapsid protein during the viral infection. The researchers tested the drug in two different mouse models: in mice that were administered plitidepsin shortly before being infected with SARS-CoV-2, the drug significantly reduced viral load (similarly to remdesivir) and lung inflammation (much better than remdesivir) compared with controls. Plitidepsin is approved for use against multiple myeloma, and its repurposing could allow the compound to directly enter phase III clinical trials against COVID-19.

These results once more certify that sea organisms/micro-organisms are incredibly prolific sources of bioactive secondary metabolites, and they are a strong encouragement for the marine natural product scientific community to continue in the efforts to exploit this unique resource.

## Figures and Tables

**Figure 1:**
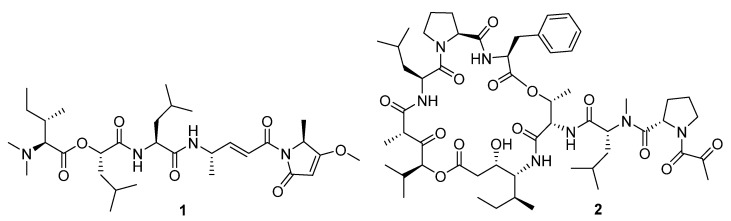
The chemical structures of gallinamide A (**1**) and plitidepsin (**2**).
